# Stroke care in the air: a new horizon for helicopter emergency medical services

**DOI:** 10.1186/s13049-025-01507-y

**Published:** 2025-12-16

**Authors:** Maren Ranhoff Hov, Karianne Larsen

**Affiliations:** 1 Department of Neurology, Oslo University Hospital, Oslo, Norway; 2 The Norwegian Air Ambulance Foundation (NAAF), Oslo, Norway; 3 Department of Prehospital Medicine, OsloMet – Oslo Metropolitan University, Oslo, Norway

Acute stroke management demands timely and accurate assessment to optimize patient outcomes. Over the past decades considerable effort has been made to develop advanced care and utilization of the prehospital services for managing acute stroke patients. However, the specific role of Helicopter Emergency Medical Services (HEMS) in acute stroke care remains insufficiently documented and poorly understood. A recent publication in the *Scandinavian Journal of Trauma, Resuscitation and Emergency Medicine* (SJTREM) [1] offered valuable insights into the application of German HEMS for interhospital transfers of stroke patients. Current guidelines emphasize the importance of prenotification and structured triage systems to optimize the chain of stroke survival, [2] and mobile stroke units (MSU), which are CT equipped ambulances, are recommended in European guidelines for prehospital management of acute ischemic stroke patients. The study in SJTREM presented a descriptive overview of stroke patients undergoing interhospital HEMS transfer, highlighting the challenges associated with HEMS in this context. The need for research of advanced diagnostic modalities, optimal staff configurations, and a broader role of HEMS in stroke management was underscored.

The study analyzed 179,000 missions conducted by 29 German air rescue helicopters, staffed by prehospital emergency physicians, paramedics, and pilots. Utilizing the national HEMS database, the researchers analyzed data from 7,528 confirmed stroke patients who underwent interhospital transfer from primary hospitals to comprehensive stroke centers between 2012 and 2022.

Although the interhospital transfer times illustrates significant time savings in favor of HEMS (21 min versus 45 min), the total transportation time from alert to arrival receiving hospital was only a median (IQR) 3.4 (IQR –14.1 to + 7.3) minutes shorter with HEMS. While it may seem intuitive that HEMS transfers are always faster, this was not the case for approximately 40% of the cohort where Ground Emergency Medical Services (GEMS) proved shorter; bearing in mind that the GEMS times were estimated based on algorithms and the time calculations did not include important metrics like handover time. Nevertheless, multiple studies across different geographic regions suggest the superiority of HEMS over GEMS transportation, especially in rural and semi-rural areas with extended travel times. HEMS has been associated with better long-term functional outcomes for LVO patients undergoing interhospital transfer, even independently of transportation time [3]. In a meta-analysis of 8 HEMS versus GEMS studies analyzing both prehospital and inter-hospital transfers, overall poor neurological outcomes were significantly reduced in the HEMS group, and HEMS was also associated with better neurological outcomes [4].

The median aerial distance between hospitals in this study was 53 km, which is a distance approximating the minimum distance required for HEMS to be more time efficient than GEMS [3].

However, rapid HEMS transportation constitutes only *one* aspect of efficient stroke care. More than 80% of the patients were critically ill (NACA ≥ 4) and 13% were intubated requiring advanced prehospital medical support which the HEMS team secures. Prior studies have provided improved outcomes for stroke patients transported by HEMS compared to GEMS without time being the reason. One can speculate if the improved outcomes can result from advanced medical support provided by the HEMS team or other external factors in-flight could be beneficial (vibrations, altitude etc.). The study does not inform on proportion ICH vs AIS, and how many received bridging tPA therapy.

In countries or regions with vast distances, remote areas and austere geography, HEMS is an indispensable resource. A road Mobile Stroke Unit (MSU) study in Norway was the first model to use solely prehospital personnel with a HEMS physician on-board the MSU. The MSU was staffed with a 3-person crew similarly to the Norwegian HEMS with an anesthesiologist, and two paramedics, and demonstrated significantly shorter overall treatment times (OTT), higher thrombolysis rate (81% vs. 59%), and no safety concerns [5].

The road MSU concept has been proposed to be integrated into rotor- and fixed-wing air ambulances, Air-MSU, not only for ultra-early stroke management, but to address disadvantages in acute stroke management for patients in rural and remote regions. However, conventional CT technology is not compatible with use in air ambulances due to its size, weight, radiation, and sensitivity to vibration and G-forces. Hence, HEMS's current contribution in acute stroke still lies in advanced life support capabilities and rapid transport to definitive care. There is great potential in HEMS in both prehospital and interhospital transfers, for acute treatment, diagnostic, monitoring and research opportunities. There are today no implemented diagnostic tools in HEMS which can elect patients to acute revascularization treatment, but new initiatives are developing towards lighter and more accessible tools.

Approximately 30% of patients received no medical intervention during transport and this raises questions about optimal triage and resource allocation. No information is provided about prior decision factors for activating HEMS, and there is a need for refined prehospital triage algorithms to ensure HEMS resources are allocated to the patients most likely to benefit. Future research should focus on geospatial analysis and the development of evidence-based algorithms for HEMS dispatch criteria in acute stroke.

Data from studies like the German HEMS provide critical insights into the challenges faced at both the organizational and patient levels. To plan for new studies exploring new technical devices for implementation in the air ambulance service and the continued need for innovation in air-based stroke interventions. Future developments should focus on integrating advanced diagnostic tools, including telemedicine, point-of-care testing, and neuroimaging, into HEMS operations. Such innovations could facilitate more precise patient assessment in the air, ensuring that the right patients receive the appropriate level of care.

Stroke care remains one of the most time-sensitive diagnoses in emergency medicine. And there is a large potential of HEMS to reduce transportation times, extend coverage to remote areas, and render advanced prehospital emergency care. Current evidence primarily derives from retrospective studies, and there is a need for real-life prospective studies also in HEMS. The expanding technological landscape—particularly in diagnostics, telemedicine, and triage algorithms—paves the way for more precise, efficient, and patient-centered air-based stroke management.

To fully realize this potential, future research should focus on prospective clinical studies including new technologies, cost–benefit analyses, geospatial analysis and the integration of hybrid models such as air-based diagnostics combined with rural diagnostic stations or MSUs. Flying advanced stroke care is within reach, and with continued innovation, it will play an increasingly important role in the global effort to improve outcomes for stroke patients.

## Data Availability

Not applicable.

## Abbreviations

AIS: Acute ischemic stroke
CT: Computed tomography
GEMS: Ground Emergency Medical Service (ambulance)
HEMS: Helicopter Emergency Medical Service
ICH: Intra cerebral hemorrhage
LVO: Large vessel occlusion
MSU: Mobile Stroke Unit
NAAF: Norwegian Air Ambulance Foundation
NACA: National Advisory Committee for Aeronautics
OTT: Onset to treatment time
SJTREM: Scandinavian Journal of Trauma, Resuscitation and Emergency Medicine
